# Peridental Inflammation

**Published:** 1864-11

**Authors:** G. A. Mills


### PERIDENTAL INFLAMMATION.

Read before the Brooklyn Dental Association, Nov. 2, 1864,

BY G. A. MILLS.

Gentlemen: I do not write on this subject thinking to educate any of you present, but simply to find out how much I know of it myself.

We will all agree that it does truly exist, and that we all come seriously in contact with it, far oftener than we could wish, hence, the great necessity of a well understood practice. The inquiring mind first asks, what is Peridental Inflammation? It is inflammation of the Periosteum. Next, what is the meaning of Periosteum? Peri, means around, osteo, bone; around the bone; Periosteum Dentinum, pertaining to the teeth. Inflammation means febrile heat, partaking of fever. Thus we have the meaning of Peridental Inflammation. It is a fever heat of the membraneous envelope of the fangs of the teeth.

What is this envelope or periosteum, its nature, etc. It is a tough fibrous membrane which adheres to nearly every part of the bone, excepting at the cartilaginous extremities where strong tendons are attached. Is highly vascular, *i. e.*, full of vessels, passing into minute orifices, which cover the entire surface of the bone. It consists of minute layers closely connected, the outer formed chiefly of connective tissue, and occasionally a few fat cells, the inner one, of elastic fibers of the finer kind, forming a dense elastic membraneous net work, superimposed, *i.e.*, laid on in several layers. In young bones it is thick and very vascular; later in life, thin and less vascular, more closely connected, adhesion growing stronger as age advances. It also serves for a nidus or nest for the ramification or division of the vessels, previous to the distribution in the bones, making the liability of foliation, or necrosis, or death of the bone, where it is denuded or stripped of this membrane, or envelope, which happens from various causes.

Peridental Inflammation arises from disturbances, with some of which we may all be familiar, and others not so; for instance, from a disturbed state of constitutional health, impaired digestion, mental excitement, pregnancy, conditions of the hard structure, etc. The Dentist generally speaking meets with Peridental Inflammation from decomposition of the tooth, pressure of decomposed matter upon the pulp, the susceptibility of the pulp to impressions of heat and cold, caused by different kinds of metallic filling, and by a thin covering of bone, caused by mechanical abrasion or by absorption, or of any of the devitalizing medicines used for the destruction of pulps, fillings over devitalized pulps not removed, (nor even an attempt made to remove them,) and by broach fillings, etc.; many of these causes *I* have met with in my practice. To understand *all* the reasons, the Dentist should be familiar with constitutional causes, then he will be able to fully diagnose any case that presents itself. The inability of the dentist to diagnose cases presenting themselves, is no doubt why we are so often defeated in our attempts to allay this inflammation, that we are unable to do so, is fully true, with many of us, many cases presented are very simple, and the causes are so plain, that we cannot err with an ordinary intelligence of dentistry, and we set about immediately removing the causes and the inflammation subsides.

If every dentist would make an honest use of the knowledge he has in possession, he would err less in his practice than he does; common sense (so very destitute in some of the first families) is so often put at bay, and we are led deep into the mire, and then we call aloud for aid; then the question is put by the suffering patient, what is the cause? and in reply, often a very unintelligent reason is assigned, covered over with a long list of technical phrases which the patient knows little of and the dentist less.

A great deal can be said relative to the causes of Peridental Inflammation, but farther I will not attempt to lead you at this time. There are wiser heads that can teach you, and have willing hearts. I will now give you some of my experience in my practice. I have met with Peridental Inflammation mostly originating from an attempt to destroy pulps that have been destroyed by causes such as these; filling over dead pulps not removed, etc. I find that great danger exists in our treatment of low toned teeth, where the necessity arises for the destruction of the pulp, both in the teeth of the young and adults, more especially the former. Once it took me (or I thought it did *from my teaching*) from seven to ten days to destroy a pulp, making an application of arsenious paste with morphia and creosote each day, and each day hooking at the delicate structure with a savage force, sometimes working on without fatigue, not being satisfied with all the pulp the tooth contained, but carrying my researches beyond the apex of the tooth or fang. Gentlemen, when I review the past I am forced to cry out, O wretched man that I have been; and yet I verily thought I was doing God service, simply from want of a primary dental knowledge, I was led astray by the ambitious holding up to me, as they did, a lucrative practice with no *Christ* in it. I hope no one here has a corresponding experience. I thank God from the bottom of my poor heart, that I was not allowed to continue such a devilish practice long. I think God took pity on my poor intelligence, and showed me my uncleanliness, for my eyes were opened, and I *did* see, as if by intuition, or inspiration, that I was an *M. D.*, miserable dentist. I have since tried to mend my ways, and I think I have made some progress. From such a practice as I first pursued, I lost nearly all the teeth that I attempted to save; caused Peridental Inflammation, and then Alveolar Abscess, principally bad practice. Remedy for the cure of such cases was specific, immediate extraction. With my present mode of practice I meet with Peridental Inflammation far less than I did formerly. One application of arsenious paste (with the same additions as named in the above formula) left in the tooth from one to three weeks, completes the destruction of the pulp, and at the expiration of this time, the pulp generally is easily removed and filled at one sitting, with no inconvenience, so far as heard from, with but few exceptions, and these are almost always confined to low toned teeth, spoken of above. And here comes the difficulty with the same kind of treatment, and very different results. Pulps destroyed, no pain, the tooth filled from the apex to the surface of the crown, patient dismissed, bill paid. No. 2 takes the chair, goes through a similar operation, passes out like the first. The patients and the operation pass out of your mind until you are called to face both under the most painful circumstances. You see at a glance that Miss A. by the expression of her face, is suffering, and she begins to unfold the history of the tooth since it was filled; perhaps it is done with kindness, and perhaps not; sometimes a terrible charge upon your intelligence is made, with the interrogatory, what should make it pain me so? and I wish I had had it out, and Miss so-and-so had had several treated in the *same* way, by Dr. A or B, and they have never given her any trouble at all. If you can convince the patient that you are "au fait" *well*, if not a skedaddling commences.

Now the inflammation may be incipient or commencing, but owing to constitutional disturbances, rapid progress is being made. The patient wishes to know what is to be done. The question now arises with the dentist at this juncture, am I sufficient for the case? What all would wish to be able to say is, I *can* effect a cure, but *all* can not say it in truth; for myself I have success and failure, but of late more success then otherwise. Numerous remedies are prescribed by A, B, and C. *but* of the number what will best relieve the case. A high fever is in rapid progress, a terrible pressure is at hand, these numerous little vessels are being taxed to the utmost; relaxation is the cry, what will produce it? Will constitutional treatment do it? Yes. Will local treatment? Yes, sometimes, and at other times, both are required. If you know of a truth what you affirm, the first thing you do is to get the full control of your case, unless you do you will work against hope. Peridental Inflammation in the beginning I have arrested by producing *nausea*, and by a *speedy* movement of the bowels. A patient of a strong bilious temperament will find more speedy relief from this treatment. I have noticed that one of scrofulous inclinations does not yield so readily to the treatment. I have in one case of this disease made a rapid change by a prescription of seidlitz powders. One case that came under my care was completely removed out of my hands, and a cure effected up to this date (it being some seventeen months since it took place) by the patient leaving town suddenly and eating a late supper of lobster salad, bringing on a severe attack of cholera morbus before morning. But I fully believe from my experience that in eight cases out of ten, that the simple application of *ice* will produce a speedy recovery, and effect a cure. Apply shaved ice if convenient in a thin piece of muslin, to the diseased parts, or apply the ice directly upon the parts, taking care not to connect with the tooth. Apply gently for a few moments, then remove for a few moments, and so on alternating. I believe there is nothing like it, and it is the easiest remedy that can be applied, and the least objectionable, and often attended with the best results.

Another remedy I have used with success: 2 parts chloroform, 1 part of camphor, mixed and applied to the diseased parts. It is cooling at first and scattering, producing after a few applications an external irritation, and effecting a cure. I have also applied leeches with some success, but attended with a good deal of inconvenience, and with some prejudice by the patient.

If Peridental Inflammation could be brought to our notice while in the incipient stage, with the remedies noticed, we should see far less of Alveolar Abscess. But how very many times do the patients take the case completely out of our hands, and we know nothing of it, except in an indirect way, as I did myself of a case but a few days since. A lady in my chair told me that Mrs. B. looked upon me as a rascal, that I filled a tooth for her some tlme since, and it gave her great trouble, ending with the lockjaw. Dr. So-and-so, of Grand Street, New York city, telling her that such was the fact, here moved the tooth and saved her life. I consoled myself with a prayer of thanksgiving that *God* had permitted so *feeble* an instrument to save the life of this most estimable lady, she now employs a Doctor of the same stamp in Brooklyn, and has no trouble.

Gentlemen, "it is not always the wise that are chosen." What we do with these cases of Peridental Inflammation must be done in earnest and in season, delays do not necessarily bring defeat, but cause much suffering and trouble to the patient and the dentist. I find by experience that delays in dentistry are dangerous as in other matters.

I could say more upon the advanced stages of Peridental Inflammation, but time will not now permit; and now, gentlemen, if I may have been instrumental in this feeble paper in throwing any light upon any mind in reference to the meaning or nature of this subject or of the manner of treatment, I shall be fully repaid for the time I have spent; but if even here I have not succeeded, I have the comforting assurance that I have done what I could.

